# Embryo-derived trypsin-induced calcium entry is inhibited by endometrial infertility factor, LEFTY2

**DOI:** 10.3389/fcell.2025.1499339

**Published:** 2025-05-29

**Authors:** Zhiqi Yang, Jing Yan, Steffen Kull, Md. Alauddin, Sara Y. Brucker, Melanie Henes, Florian Lang, Madhuri S. Salker

**Affiliations:** ^1^ Department for Women’s Health, University Hospital Tübingen, Tübingen, Germany; ^2^ Department of Physiology, University of Tübingen, Tübingen, Germany; ^3^ Department of Physiology, Jining Medical University, Jining, China

**Keywords:** LEFTY2, Ca^2+^ channels, endometrium, receptivity, unexplained reproductive failure

## Abstract

**Introduction:**

A transient window of uterine receptivity ensures that embryos implant in an optimal endometrial environment. Failure to establish or premature closure of the implantation window is thought to be a major cause of infertility, which affects many couples globally. Embryos release trypsin, which designates its developmental potential and plays a crucial role in implantation. Calcium (Ca^2+^) signalling participates in receptivity and is thus a prerequisite for embryo implantation. Left-right determination factor 2 (LEFTY2) is a negative regulator of endometrial receptivity and is associated with unexplained infertility. We hypothesize that LEFTY2 impedes Ca^2+^ entry induced by trypsin in endometrial cells.

**Methods:**

*In silico* analysis was performed to investigate classical trypsin pathway genes in human embryos. Trypsin levels from single human embryo conditioned medium were subject to ELISA. To determine if trypsin signals can modulate calcium entry, intracellular calcium [Ca^2+^]_i_ was determined utilizing Fura-2 fluorescence in human endometrial epithelial cells (Ishikawa cells). Bioinformatic analysis on publicly available single cell sequencing data was used to investigate the expression of *L-type calcium channel (CACNA1C)* in endometrium. qRT-PCR and immunofluorescence were used to quantify L-type calcium channel abundance.

**Results:**

We report that the trypsin machinery is established at the blastocyst stage and that high levels of trypsin are associated with a successful pregnancy. Treatment with LEFTY2 or combined treatment with LEFTY2 and trypsin blocked the increase of L-type Ca^2+^ channel levels and activity. Treatment of endometrial cells with trypsin was followed by an increase of [Ca^2+^]_i_, an effect that was significantly blunted by amiloride and LEFTY2. Further, the trypsin induced increase of [Ca^2+^]_i_ was significantly blunted by L-type calcium channel inhibitor nifedipine. In the presence of nifedipine, LEFTY2 did not further modify trypsin induced increase of [Ca^2+^]_i_. LEFTY2 significantly decreased levels of L-type Ca^2+^ channel.

**Discussion:**

Taken together, we demonstrate that high trypsin levels are associated with a positive pregnancy outcome and that infertility factor LEFTY2 downregulates trypsin induced Ca^2+^ increase due in part by interference with nifedipine sensitive Ca^2+^ entry. These findings contribute further to our knowledge of unexplained infertility and failed assisted reproductive technologies.

## 1 Introduction

Human reproduction is often considered surprisingly inefficient, echoed by the evidence that only 40%–60% of conceptions in young, healthy women result in a successful birth (Muter et al., 2023). The causes of female infertility are vast ranging from endometriosis, ovulation and fallopian tube disorders (Ehsani et al., 2019). Furthermore, in many industrialised countries, factors such as obesity, metabolic syndrome, vaping/smoking and advanced maternal age (AMA) also contribute to infertility (Emokpae and Brown, 2021). Despite these identifiable factors, approximately 30% of infertility cases remain unexplained (Dougherty et al., 2023). Failure of the endometrium to achieve a receptive state is thought to be a major cause of infertility and the rate-limiting step in assisted reproductive technology (Gellersen and Brosens, 2014). Furthermore, large clinical studies revealed that the implantation rate drops significantly in women with reduced fertility, even when using donor oocytes from young women pointing to a critical role of the endometrium or aberrant endometrial factor(s) as the key culprit (Toner et al., 2002; Soares et al., 2005; Loid et al., 2024). However, the mechanisms underlying these pregnancy failures are poorly understood.

Decidualization is the transformation of endometrial stromal fibroblasts into specialized secretory decidual cells and is critical for establishing a supportive environment essential for embryo implantation and subsequent placental development (Gellersen and Brosens, 2014). In humans, decidualization occurs in the mid-luteal phase of the menstrual cycle and occurs independently of pregnancy (Okada et al., 2018). In each menstrual cycle, the endometrium undergoes estrogen-driven proliferation followed by progesterone-induced differentiation, resulting in a brief window during which embryo implantation can occur. In humans, this receptive window opens approximately 6 days after the pre-ovulatory LH surge and lasts for about 4 days, typically aligning with days 19–22 of a typical 28-day menstrual cycle (Muter et al., 2023). The decidual micro-environment is dynamic and capable of responding and adapting to embryo-cross talk, and it is therefore proposed that the endometrium can act as a ‘bio-sensor’ of embryo quality. Interference by extrinsic factors or ‘malfunctioning’ of the maternal endometrial biosensor may lead to an inhibition of implantation or out-of-phase implantation of non-viable embryos known as the ‘selection hypothesis’ (Weimar et al., 2012; Teklenburg et al., 2010a). This notion has been further corroborated by the observation that endometrium from women suffering with unexplained infertility or recurrent implantation failure do not respond to intrinsic and embryonic cues (Simon and Laufer, 2012).

For the endometrium to function as a biosensor, human embryos must produce mechanical or chemical signals that convey their developmental potential to the maternal cells. Human blastocysts release various signals such as interleukins, microRNAs, mucins, growth factors, hormones, and trypsin-like proteases, which designate their developmental potential and play a role in the implantation process (Wang and Dey, 2006; Ruan et al., 2012; Brosens et al., 2014). Trypsin-like proteases are known to play a role in early embryo development in invertebrates and vertebrates, including mouse and rhesus monkey (Mishra and Seshagiri, 2000; Perona and Wassarman, 1986; Lin et al., 2006; Jiang et al., 2011). Trypsin, a serine protease secreted by the murine blastocyst, has been implicated in cross-talk with the uterine epithelium (Shmygol and Brosens, 2021). According to studies of Ruan et al. in a murine model, trypsin (released by embryos) cleaved the α-subunit epithelial sodium channel (ENaC) present on epithelial cells, leading to cell membrane depolarization. This depolarization activated the L-type voltage gated Ca^2+^ channel, resulting in a sustained cytosolic Ca^2+^ activity ([Ca^2+^]_i_) rise. The increase in [Ca^2+^]_i_ resulted in a cyclooxygenase 2 (COX2) dependent rise in prostaglandin E_2_ (PGE_2_) release, thereby augmenting the process of implantation and decidualization (Ruan et al., 2012). Furthermore, in mice, using either a serine protease inhibitor or amiloride (ENaC inhibitor) was found to reduce the number of implantation sites (Sun et al., 2007).

Left-right determination factor 2 (LEFTY2) or endometrial bleeding associated factor (EBAF) is a member of the transforming growth factor (TGF)-β superfamily (Cornet et al., 2002; Ulloa and Tabibzadeh, 2001). LEFTY2 is initially produced as precursor, which is then cleaved to release the C-terminal monomeric active protein (Tabibzadeh and Hemmati-Brivanlou, 2006). LEFTY2 is highly expressed in decidualizing human endometrial stromal cells (HESCs) during the late luteal phase of the menstrual cycle, coinciding with the closure of the window of implantation (Gellersen and Brosens, 2014; Tabibzadeh et al., 1998). Enhanced LEFTY2 expression was associated with unexplained infertility (Salker et al., 2011), abnormal uterine bleeding (Tabibzadeh and Kothapalli, 1996; Kothapalli et al., 1997) and implantation failure (Tabibzadeh et al., 2000; Tang et al., 2005). Further, *in vivo* gene transfer of LEFTY2 in the mouse uterus led to implantation failure, though the mechanism remains to be defined. The present study determined trypsin activity from single human embryo conditioned media and tested whether infertility factor LEFTY2 can modify trypsin-induced Ca^2+^ entry in a model of human endometrial epithelial cells.

## 2 Materials and methods

### 2.1 Cell culture

Human endometrial epithelial cells (Ishikawa cells; ECACC-99040201; a widely used model for implantation) (Brosens et al., 2014; Jiang et al., 2017; Tang et al., 2018; Kumar et al., 2017; Salker et al., 2016a) were maintained in Dulbecco’s modified Eagle’s medium/F12 without phenol red (Invitrogen, Germany) supplemented with fetal bovine serum (FBS, Gibco, Germany), 1% (v/v) antibiotic-antimycotic solution (Gibco, United States), and 0.25% (v/v) L-glutamine (Gibco, United Kingdom). Cells were incubated at 37°C in a humid atmosphere maintained at 5% (v/v) CO_2_. Cells were tested for mycoplasma infection at regular intervals. Cells were normally seeded at 2 × 10^5^ and allowed to recover for 24 h. Where indicated, the cells were treated with LEFTY2 for 6 h (25 ng mL^−1^; R&D Systems, Germany) as previously described (Salker et al., 2016a; Salker et al., 2015; Salker et al., 2016b) and/or trypsin for 24 h (Ruan et al., 2012) (20 μg mL^−1^; Invitrogen, Germany) in the absence and presence of the ENaC inhibitor amiloride (1 μM; Sigma, Germany), or L-type Ca^2+^ channel inhibitor nifedipine (10 μM; Sigma, Germany) for the indicated periods and with the indicated concentrations. To mimic the *in vivo* decidualization environment, the cells were treated with 0.5 μM 8-Bromo-cAMP (cAMP, Tocris, United Kingdom) and 1 μM Medroxyprogesterone 17-acetate (MPA, Sigma, Germany) for 6 days as described in the previous study (Salker et al., 2018), followed by treatment with LEFTY2 and trypsin as described above.

### 2.2 Ca^2+^ measurements

Fura-2 fluorescence was used to determine intracellular Ca^2+^ activity (Bhavsar et al., 2013). Cells were incubated with Fura-2/AM (2 μM, Invitrogen, Germany) for 20 min at 37°C. SOCE was determined by extracellular Ca^2+^ removal in the presence of sarco/endoplasmic Ca^2+^ ATPase inhibitor thapsigargin (1 μM, Invitrogen, Germany) and subsequent Ca^2+^ re-addition. For sodium-calcium exchanger (NCX)-induced calcium entry, extracellular Na^+^ was removed by replacing it with Li^+^ or Choline, promoting calcium influx through NCX. For potassium-dependent sodium-calcium exchanger (NCKX)-induced calcium entry, both extracellular Na^+^ was removed and extracellular K^+^ was added to enhance NCKX activity. Cells were excited alternatively at 340 nm and 380 nm through an objective (Fluor 40×/1.30 oil) built on an inverted phase-contrast microscope (Axiovert 100, Carl Zeiss, Germany). Emitted fluorescence intensity was recorded at 505 nm. Data were acquired using specialized computer software (Metafluor, Universal Imaging, United States). Cytosolic Ca^2+^ activity was calculated from the 340 nm/380 nm ratio (Bhavsar et al., 2013; Yang et al., 2014).

### 2.3 Flow cytometry

Ishikawa cells were treated as described in above. The trypsin-induced calcium entry was estimated by flow cytometry using Fluo-4 staining (F14201, Invitrogen, Germany) in accordance with the manufacturer’s instructions. Briefly, cells were collected by trypsin and washed with PBS, then suspended in calcium- containing PBS (1 mM CaCl_2_ and 0.49 mM MgCl_2_) with 5 µM Fluo-4. After that, cells were then incubated at room temperature for 40 min, protected from light, and washed again with calcium-containing PBS, and analysed by flow cytometry. Data were analysed using the Flowjo software (Flowjo LLC, Oregon, United States).

### 2.4 Quantitative real time-PCR (qRT-PCR)

Total mRNA was extracted from whole cell cultures using Trizol (Invitrogen, Germany) followed by the phenol-chloroform protocol. 2 μg of mRNA was reverse transcribed using the Maxima™ H Minus cDNA Synthesis Master Mix with dsDNase (M1681, ThermoFisher Scientific, Germany), following the manufacturer’s protocol. The resulting first-strand cDNA was diluted and stored at −20°C. Primers were designed using the NCBI, PrimerBlast software. Human ribosomal protein L19 (L19; RPL19) was used as the endogenous housekeeping gene, to normalize for variances in input cDNA. Primer sequences will be provided on request. Detection of gene expression was performed with PowerUp SYBR Green Master Mix (A25742, Thermofisher Scientific, Germany) and quantitative RT-PCR was performed on a QuantStudio 3 Real-Time PCR system (A28567, Thermofisher Scientific, Germany) using universal cycling conditions. Transcript levels were determined using the ^ΔΔ^Ct method and expressed as arbitrary units (a.u). Non-template control (NTC) reactions (cDNA was substituted with DEPC water) and reverse transcriptase (RT) controls were also included. In NTC or RT control reactions PCR products were not detected (data not shown). Melting curve analysis and agarose gel electrophoresis confirmed amplification specificity.

### 2.5 Protein extraction and western blotting

Total protein samples were prepared by lysing the adherently cultured Ishikawa cells in Laemmli buffer containing 0.5 M Tris hydrochloride (Roth, Germany) pH 6.8, 20% Sodium dodecyl sulfate (SDS, Sigma, Germany), 0.1% Bromophenol blue (Serva, Germany),1% beta mercaptoethanol (Sigma, Germany), and 20% glycerol (Roth, Germany). Whole cell protein lysates were heated at 95°C for 5 min.

Extracts were loaded on to a 12% sodium dodecyl sulfate poly-acrylamide gel (SDS-PAGE) using the XCell SureLock® Mini-Cell apparatus (Invitrogen, Germany) followed by electrophoresis. The protein from the gel was transferred onto a poly-vinylidenefluoride membrane (Amersham Biosciences, Germany). After air drying, the membranes were activated in 100% methanol and subsequently blocked using 5% non-fat milk or bovine serum albumin (BSA) for 1–2 h at RT. Membranes were probed overnight at 4°C with antibodies: LEFTY2 (1:500, sc-365845, Santa Cruz, Germany), CACNA1C (1:200, ACC-013, Alomone Labs, Israel), COX2 (1:200, MA5-14568, ThermoFisher, Germany), GAPDH (1:1,000, #2118L, Cell Signaling, Germany) was used for loading control. After 3 washes with TBS-T, each for 10 min, the membranes were incubated with HRP-conjugated anti-rabbit secondary antibody (1:2000, #7074s, Cell Signaling, Germany) or HRP-conjugated anti-mouse secondary antibody (1:2000, 7076S, Cell Signaling, Germany) at RT for 1 h, followed by 3 washes with TBS-T. Protein bands were detected using a chemiluminescent detection kit (WesternBright™ ECL, ThermoFisher, Germany) and visualized by using iBrightTM Imaging System (Invitrogen, Germany). Bands were quantified with ImageJ Software (Schindelin et al., 2012). Full uncropped Western blotting images are provided in the Supplementary Figures 1, 2.

### 2.6 Immunofluorescence

Ishikawa cells were plated on 12 mm round coverslips at a density of 5,000 cells per coverslip. Treatment was performed as described above. Post treatment the cells were fixed for 15 min with 4% paraformaldehyde (PFA, Sigma, Germany), washed 3 times with PBS, and permeabilized for 10 min in 0.1% Triton X-100 (Sigma, Germany)/PBS. The coverslips were blocked with 5% BSA (Sigma, Germany) in 0.1% TritonX-100/PBS for 1 h at room temperature and were probed overnight at 4°C with primary antibody: anti-CACNA1C antibody (1:100, ab84814, Abcam, United Kingdom). After 3 washes, coverslips were probed overnight at 4°C with secondary antibody: Alexa Fluor 568 (2 µg/mL, #A-11004, Invitrogen, Germany). The coverslips were mounted with ProLong Gold antifade reagent with DAPI (#P36931, Invitrogen, Germany) on slides. Microscopy was performed with EVOS M7000 cell imaging system (ThermoFisher, Germany) with × 20 objective. Scale bar was 25 μm.

### 2.7 Enzyme-linked immunosorbent assay (ELISA)

Ishikawa cells were treated as stated above. The culture medium was harvested and stored at −80°C. The collected cultured medium was processed for ELISA by using human prostaglandin E_2_ (PGE_2_) ELISA Kit (#KHL1701, Invitrogen, Germany) following the manufacturer’s instructions. The absorbance was measured with Varioskan LUX spectrophotometer (ThermoFisher Scientific, Germany).

### 2.8 Ethical approval

This study (218/2023BO2) was approved by the Ethics Commission at the Medical Faculty of Eberhard-Karls University of Tübingen. Written informed consent was obtained from all participating patients/parents who were attending the *in vitro* fertilization (IVF) clinic, Universitätsklinikum Tübingen. The patient sample of 24 was determined using a power calculation (G*’Power; 80% power and alpha-type 1 error of 5%) based on a previous publication (Kang, 2021).

### 2.9 Embryo conditioned media collection and trypsin activity using ELISA based methods

Individual embryos were cultured in 25 µL droplets in Sage 1-step medium (Origio, CooperSurgical, Germany) media using the EmbryoScope® (Vitrolife GmbH, Germany). Embryos graded as ‘A’ were transferred back to the recipient and retrospectively determined for a positive pregnancy up to 12 weeks after. The embryo slide was collected and the embryo media was collected immediately and frozen at −80°C until further use. Wells which contained media but with no embryo contact was used as an internal control. Trypsin activity ELISA kit (AB102531, Abcam, United Kingdom) was used and results calculated precisely according to the manufacturer’s instructions. Samples were then correlated to a positive pregnancy test.

### 2.10 Data mining

*In silico* analysis was performed on the following publicly available datasets from the Gene Expression Omnibus (GEO): Pre-implantation embryonic development (*Homo sapiens*; ID:GDS3959) (Xie et al., 2010). Bioinformatic analysis was performed on publicly available single cell sequencing data from the Single Cell Expression Atlas (Vento-Tormo et al., 2018).

### 2.11 Statistical analysis

Data were analysed with the statistical package Graphpad Prism (Graphpad software Inc). Unpaired Student’s t-test and one-way ANOVA were used where appropriate. Statistical significance was assumed when P < 0.05. Data were exported to Microsoft Excel for analysis and graphs were generated and analysed using GraphPad Prism® Software.

## 3 Results

### 3.1 Expression patterns and impact of trypsin pathway genes in human preimplantation embryos

**FIGURE 1:**
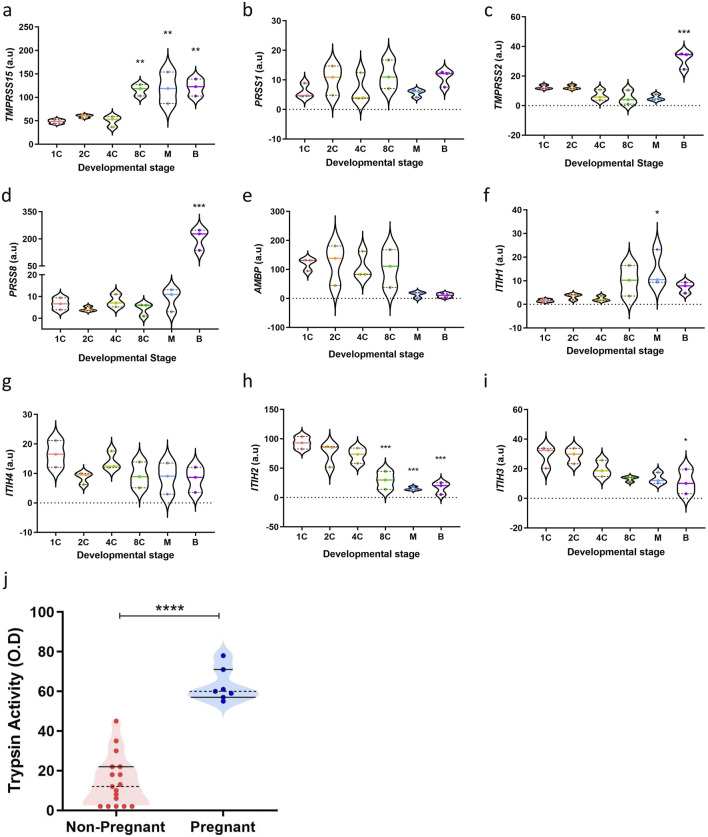
Trypsin activity increases with developmental maturation. **(a–i)** Key classical trypsin pathway genes expression in human pre-implantation embryos (n = 3) (GEO accession number: GDS3959) (1C: 1-cell embryo, 2C: 2-cell embryo, 4C: 4-cell embryo, 8C: 8-cell embryo, M: morula, **(b)** blastocyst). The data are presented as mean ± SEM. One-way ANOVA were used to calculate statistical significance. Asterisks (*P < 0.05, **P < 0.01, ***P < 0.001, ****P < 0.0001) indicate a significant difference compared to the 1-cell embryo stage. **(j)** Trypsin activity in individual droplets collected (n = 24). All measurements were performed in duplicate, normalised to unconditioned medium and are displayed as individual values. The data are presented as mean ± SEM. Unpaired Student’s t-test used to calculate statistical significance. ****P < 0.0001.

For the endometrium to act as a biosensor, the human blastocyst must signal its potential to the maternal cells. Trypsin-like proteases are known to play a role in early embryo development. Trypsin-like proteases are involved in early and pre-implantation embryo development in invertebrates such as *drosophila*, salmon, marine crab, *Xenopus* (Skern-Mauritzen et al., 2009; Misra et al., 1998; Xu et al., 2013; Yamada et al., 2000) and in vertebrates such as mouse, rat, rabbit, sheep, hamster and rhesus monkeys (Mishra and Seshagiri, 2000; Perona and Wassarman, 1986; Lin et al., 2006). Whether this plays a role in human embryos remains untested. To explore the expression patterns of genes implicated in the trypsin pathway, we mined publicly available transcriptomic data (GEO accession number: GDS3959) of human embryos at differing preimplantation developmental stages (1C; 1-cell embryo, 2C; 2-cell embryo, 4C; 4-cell embryo, 8C; 8-cell embryo, M; morula and B; blastocyst) (Xie et al., 2010). As shown in Figure 1, transcriptomic analyses of key classical trypsin pathway gene transcripts were present in human pre-implantation embryos, highlighting a mechanism of trypsin activity at the blastocyst stage. Trypsin is produced through the enzymatic breakdown of trypsinogen, and its activity can be regulated by various trypsin and protease inhibitors. Enteropeptidase, encoded by *transmembrane serine protease 15 (TMPRSS15)*, is considered the ‘master regulator’ of trypsin activity due to its role in activating trypsinogen by cleavage (Smith and Johnson, 2013). In Figure 1a, we showed that *TMPRSS15* expression significantly increases at the 8-cell stage in human embryos (P = 0.0043), whilst the *protease serine 1 (PRSS1)* gene (Figure 1b), which encodes trypsinogen, is not regulated during preimplantation development. Additionally, the highly regulated serine protease genes *transmembrane protease serine subtype 2 (TMPRSS2)* (P = 0.0004) and prostasin (*PRSS8*, P < 0.0001) are also notably upregulated at the blastocyst stage in human embryos (Figures 1c, d).

Critically, several trypsin inhibitors also play a role in regulating trypsin activity during embryo development. Alpha-1-microglobulin/bikunin precursor (AMBP) is a plasma protein that includes bikunin, which acts as a potent trypsin inhibitor *via* its Kunitz-type protease inhibitor domain (Sanchez et al., 2002). As Figure 1e showed, expression of *AMBP* decreases at the morula stage in human embryos (P = 0.1032). Inter-α-trypsin inhibitors (IαI), composed of heavy chains (ITIH1-4) and the light chain bikunin, also contribute to trypsin inhibition. The heavy chains stabilize the inhibitory function of bikunin by forming complexes with it (Lord et al., 2020; Zhuo and Kimata, 2008). The transcriptional regulation of *ITIH* genes during pre-implantation development may therefore influence embryo-derived trypsin activity. *ITIH1* expression increases beyond the 4-cell stage (P = 0.0301), while *ITIH4* expression is lowest at the 2-cell stage (P = 0.2276) (Figures 1f, g). Additionally, *ITIH2* (P = 0.0007) and *ITIH3* (P = 0.0247) expression progressively decreases beyond the 2-cell stage in human pre-implantation embryos (Figures 1h, i). These findings indicate decreasing trypsin inhibition at later stages of human pre-implantation embryo development, strengthening a role for trypsin as a vital embryonic signal at implantation.

To assess whether trypsin activity levels can serve as indicators of embryo implantation potential, trypsin activity was measured in embryo culture media (ECM) from individual day 5 single embryo transfer (SET) embryos. The criteria for SET in our unit include maternal age under 37 years, one high-quality blastocyst (the embryo having an “A” grade for both the inner cell mass and the trophectoderm) and no prior failed IVF cycles. By selecting ECM from SETs, we controlled for factors such as patient age, embryo quality, developmental stage, and prognosis, ensuring that the trypsin levels detected in the ECM were directly related to pregnancy outcomes.

ECM samples were subsequently collected and analysed for trypsin activity using an ELISA-based method. Notably, trypsin activity was found to be significantly higher in ECM from embryos that implanted following SET, compared with those that failed to implant (Figure 1j, P < 0.0001). The median absorbance was 14.65 ± 12.87 O.D. for the non-pregnant group and 63 ± 8.35 O.D. for the pregnant group, with ranges of values from 2 to 45 O.D. for the non-pregnant group and 55 to 78 O.D. for the pregnant group. Taken together, these findings confirm a role for trypsin as a potential embryonic signal and information on embryo competency.

### 3.2 LEFTY2 modulates trypsin-induced calcium influx in endometrial epithelial cells

**FIGURE 2:**
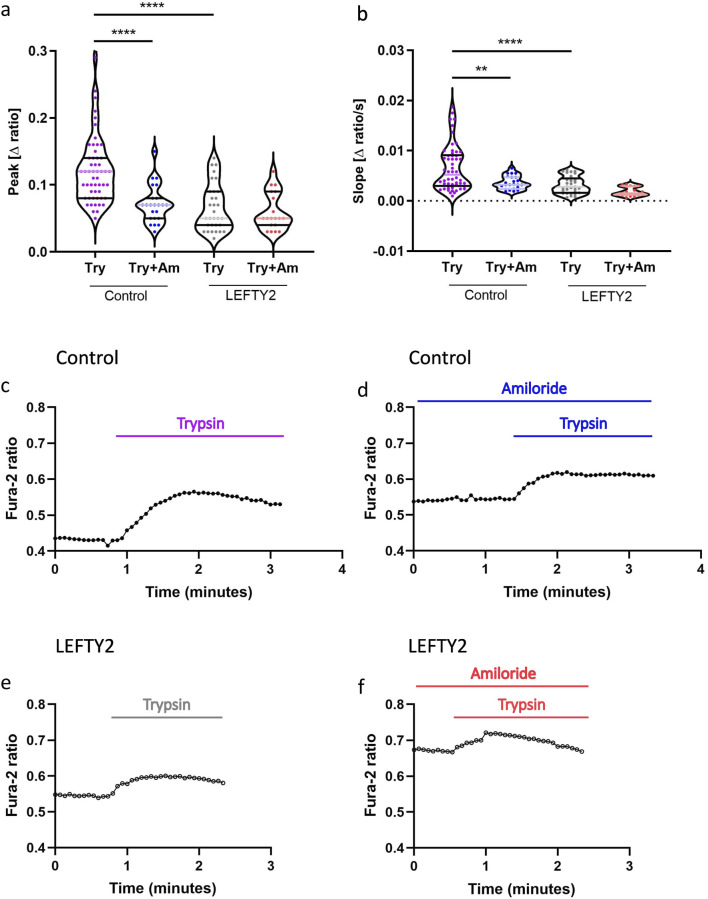
Trypsin induced Ca^2+^ entry in human endometrial epithelial cells is blocked by the presence of LEFTY2. **(a, b)** Arithmetic means (± SEM, n = 19–54 cells) of the peak value **(a)** and slope **(b)** of the change in intracellular Ca^2+^ concentrations following trypsin treatment (purple) without and with the presence of amiloride, without (left bars, Control) and with (right bars, LEFTY2) pretreatment with LEFTY2 (25 ng/mL, 6 h). **(c–f)** Representative original tracings showing intracellular Ca^2+^ concentrations in Fura-2/AM loaded human endometrial epithelial cells without (black circles) and with (open circles) pretreatment with LEFTY2 (25 ng/mL, 6 h) human endometrial epithelial cells prior to and following addition of trypsin (20 µg/mL) without **(c, e)** and with **(d, f)** the presence of amiloride (10 µM). The amplitude (peak) and the velocity (slope, calculated from the linear fit) of the Ca^2+^ entry was analysed. The data are presented as mean ± SEM. One-way ANOVA was used to calculate statistical significance. **P < 0.01, ****P < 0.0001.

Failure to establish the implantation window is thought to be a major cause of infertility. A putative candidate is LEFTY2, which we have shown to be involved in unexplained infertility (Salker et al., 2018). In the next series of experiments, we investigated the effect of trypsin on intracellular calcium ([Ca^2+^]_i_) levels and explored whether endometrial infertility factor, LEFTY2 could interfere with this process. As illustrated in Figures 2a–c, the addition of trypsin (closed circles, ●) led to a rapid increase in [Ca^2+^]_i_, characterized by a pronounced rise in both slope and peak. This trypsin-induced calcium influx was significantly attenuated by the ENaC blocker amiloride (10 µM), as shown in Figures 2a, b, d. Further analysis involved pre-treating endometrial epithelial cells with LEFTY2 (25 ng/mL, open circles ○) for 6 h, as previously described (Salker et al., 2016a). This pre-treatment resulted in a notable reduction in trypsin-induced Ca^2+^ entry, as depicted in Figures 2a, b, e. Interestingly, the presence of amiloride in combination with LEFTY2 pre-treatment showed a trend towards an even greater reduction in [Ca^2+^]_i_, though this combined effect did not achieve statistical significance, as indicated in Figures 2a, b, f.

### 3.3 LEFTY2 attenuates trypsin-induced upregulation of L-type calcium channels

**FIGURE 3:**
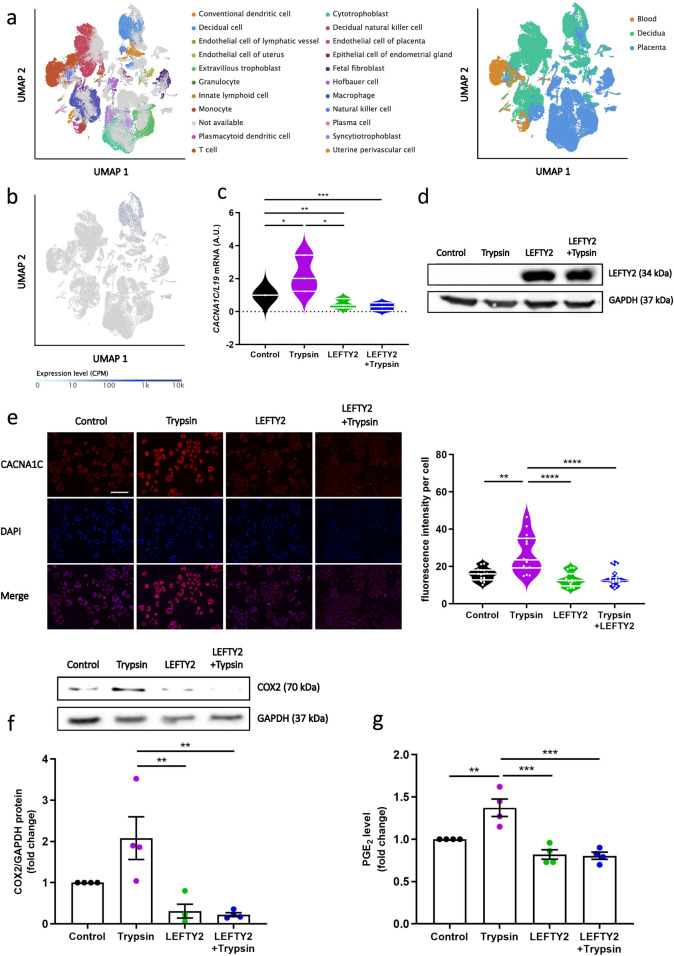
LEFTY2 decreases L-type ca^2+^ Channel abundance. Cell cultures were treated with or without LEFTY2 (25 ng/mL) for 6 h either in the presence or absence of Trypsin (20 µg/mL, 24 h). **(a)** Uniform manifold approximation and projection (UMAP) clustering of tissues and cell types; Tissue compartments and cell types were annotated in the Single Cell Expression Atlas. **(b)** Expression of *CACNA1C* in single cells, presented as counts per million (CPM), overlaid on the UMAP map. **(c)** Arithmetic means ± SEM (n = 6) of transcript levels encoding the human *CACNA1C* transcript levels were determined by qRT-PCR, normalized to the levels of *L19* mRNA and expressed in arbitrary units (a.u.). **(d)** Western blot analysis of LEFTY2 expression in Ishikawa cells (n = 4). GAPDH was used as a loading control. **(e)** IF microscopy of Ishikawa cells treated with or without LEFTY2 (25 ng/mL) for 6 h either in the presence or absence of Trypsin (20 µg/mL, 24 h) showing CACNA1C subcellular localization. CACNA1C fluorescence intensity quantification results were shown (right). CACNA1C: Alexa Fluor 568 (red); nucleus: DAPI (blue). Quantification performed from 3 experiments with >15 cells quantified for each condition. Scale bar = 25 µm. **(f)** Western blot analysis of COX2 expression in Ishikawa cells (n = 4). GAPDH was used as a loading control. **(g)** PGE_2_ level determined by ELISA. The data are presented as mean ± SEM. One-way ANOVA was used to calculate statistical significance. *P < 0.05, **P < 0.01, ***P < 0.001, ****P < 0.0001.

The previous results showed that decrease of calcium does not occur by blocking ENaC. We next tested whether the inhibitory effect of LEFTY2 on Ca^2+^ entry was paralleled by altered L-type calcium channel levels. To determine whether L-type voltage gated Ca^2+^ channel (CACNA1C) is expressed in normal human endometrial tissue, we first performed bioinformatic analysis on publicly available single cell sequencing data (Vento-Tormo et al., 2018). We observed an expression of *CACNA1C* in human decidua, particularly in endometrial decidual cells (Figures 3a, b). As shown in Figure 3c, treatment of trypsin alone increased *CACNA1C* transcript levels, in keeping with previous findings (Ruan et al., 2012). Transcript levels were reduced in the presence of LEFTY2 and following co-treatment with LEFTY2 and trypsin. The induction of LEFTY2 was confirmed by western blotting (Figure 3d). Subsequently, immunofluorescence analysis demonstrated that the intensity of cytosolic CACNA1C increased upon trypsin treatment, while it decreased following treatment with LEFTY2 and co-treatment with LEFTY2 and trypsin (Figure 3e). The original images are provided in Supplementary Figure 3. Additionally, we also showed the same trend by western blot upon the co-treatment of LEFTY2 and trypsin (Supplementary Figure 4). Furthermore, to investigate the downstream factors regulated by trypsin-induced Ca^2+^ entry, we measured COX2 levels using Western blotting. As shown in Figure 3f, COX2 protein expression increased following trypsin treatment and decreased after treatment with LEFTY2. Since COX2 is a key enzyme in PGE_2_ synthesis, we further measured PGE_2_ levels using ELISA. As shown in Figure 3g, PGE_2_ levels followed the same pattern as COX2 under the same treatment conditions.

### 3.4 LEFTY2 and nifedipine interaction in modulating trypsin-induced calcium entry

**FIGURE 4:**
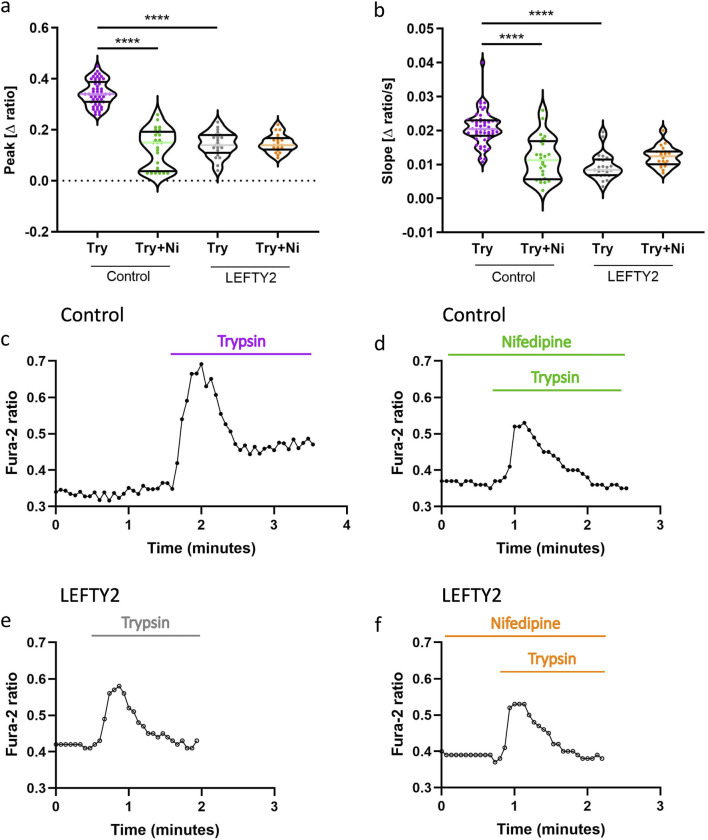
LEFTY2 decreases the trypsin induced Ca^2+^ entry by impeding the nifedipine sensitive L-type calcium channel. **(a, b)** Arithmetic means (± SEM, n = 19–54 cells) of the peak value **(a)** and slope **(b)** of the change in intracellular Ca^2+^ concentrations following trypsin treatment without and with the presence of nifedipine, without (left bars, Control) and with (right bars, LEFTY2) pretreatment with LEFTY2 (25 ng/mL, 6 h). **(c-f)** Representative original tracings showing intracellular Ca^2+^ concentrations in Fura-2/AM loaded human endometrial epithelial cells without (black circles) and with (open circles) pretreatment with LEFTY2 (25 ng/mL, 6 h) human endometrial epithelial cells prior to and following addition of trypsin (20 µg/mL) without **(c, e)** and with **(d, f)** the presence of nifedipine (10 µM). The nifedipine (peak) and the velocity (slope, calculated from the linear fit) of the Ca^2+^ entry was analysed. The data are presented as mean ± SEM. One-way ANOVA was used to calculate statistical significance. ****P < 0.0001.

To evaluate whether the inhibitory effect of LEFTY2 on trypsin-induced Ca^2+^ entry is sensitive to nifedipine, a known L-type calcium channel blocker, we conducted experiments with trypsin in the absence and presence of nifedipine (10 µM) (Manohar et al., 2021). As shown in Figures 4a–d, both slope and peak of the [Ca^2+^]_i_ increase induced by trypsin (closed circles, ●) were significantly attenuated when nifedipine was present. This indicates that nifedipine effectively blunts the trypsin-induced upregulation of [Ca^2+^]_i_. Additionally, the pre-treatment with LEFTY2 (25 ng/mL, open circles ○) resulted in a notable reduction in trypsin-induced Ca^2+^ entry, as depicted in Figures 4a, b, e. However, when LEFTY2 was applied in the presence of nifedipine, it did not significantly alter the trypsin-induced Ca^2+^ increase, as depicted in Figures 4a, b, f. These results suggest that the inhibitory action of LEFTY2 on Ca^2+^ entry is at least partially mediated through pathways sensitive to nifedipine.

## 4 Discussion

Unexplained infertility presents a major challenge for reproductive medicine professionals. Reduced endometrial receptivity during the implantation window for embryos may be a key factor contributing to unexplained infertility and failed IVF cycles (Stevens et al., 2022). It has been shown that the endometrium can act as a ‘bio-sensor’ of embryo quality, in order to limit maternal investment of non-viable embryos (Teklenburg et al., 2010b; Macklon and Brosens, 2014). The ‘selection hypothesis’ suggests that an excessive or pronounced decidual response can shorten the window of receptivity and enhance the elimination of embryos, thereby lowering the risk of miscarriage but potentially preventing conception (Brosens et al., 2014). Therefore, the human uterus has an intrinsic ability to adapt and can adjust its receptivity and selectivity traits (Gellersen and Brosens, 2014).

Embryos communicate *via* serine proteases including trypsin, as demonstrated in mammals where blastocysts secrete proteases to induce calcium signaling in endometrial cells during implantation (Hennes et al., 2023) and in *Drosophila*, where serine proteases like *Easter* are involved in patterning by activating a proteolytic cascade (Misra et al., 1998). Trypsin is formed through the enzymatic cleavage of trypsinogen and is primarily regulated by enteropeptidase, which serves as regulator by activating trypsinogen through cleavage (Zheng et al., 2009; Whitcomb et al., 1996; Szabo et al., 2003; Hegyi and Sahin-Toth, 2017). This initiation causes trypsin release, which is able to auto-activate trypsinogen thereby increasing trypsin production (Szabo et al., 2003). The gene encoding trypsinogen itself, *PRSS1*, is not regulated during pre-implantation development and the gene encoding *PRSS8*, located on chromosome 16 is a serine protease expressed highly by healthy embryos. Interestingly, chromosomes 16 and 22 are the most common chromosomes affected by trisomy (Munne et al., 2004). The augmented gene dosage in trisomic embryos could result in an over-dosage of embryonic trypsin thereby encouraging invasion of the chromosomally ‘abnormal’ embryo (Quenby et al., 2002; Ma et al., 2009). In this study, we show that trypsin activity was measurable in ECM from individually cultured human embryos and was found to be related to successful pregnancies. A limitation of our study was that the participating patients were selected based on their infertility. As a result, the generalizability of the findings to a broader population, including those without known fertility issues should be assessed. Further research involving a more diverse patient cohort is necessary to validate the observed association between trypsin activity from ECM and successful pregnancies to rule out the effects of genetics and ancestry. Further refinement of this analysis could provide a novel complementary approach to embryo grading and selection in IVF treatment by incorporating embryo-derived trypsin as a potential marker of implantation, alongside traditional morphology-based assessments.

ENaC, located on the epithelium, has been shown to be upregulated during the peri-implantation period in mice (Liu et al., 2014) where it controls water and electrolyte resorption and is known to contribute to uterine closure in mice (Li et al., 2023). ENaC is activated by a variety of mechanical stimuli, such as changes in shear stress or stretching of the epithelial tissue (Kleyman et al., 2018) and by serine proteases, which cleave specific segments of the channel to facilitate its activation (Shi et al., 2013). Serine proteases, including trypsin, are present at the embryo-endometrial interface, where they are released by the embryo and are essential for successful implantation (Brosens et al., 2014; Salamonsen and Nie, 2002). As previously proposed, trypsin likely facilitates its effects through the activation of ENaC, resulting in Na^+^ influx and subsequent membrane depolarization (Ruan et al., 2012).

Further, our present observations reveal that endometrial infertility factor LEFTY2 downregulates trypsin-induced Ca^2+^ entry. Treatment of endometrial epithelial cells with trypsin was followed by a rapid increase of [Ca^2+^]_i_, an effect that was significantly blunted by ENaC inhibitor amiloride. We acknowledge the limitations of using an *in vitro* model that uses a human carcinoma-derived endometrial epithelial cells as the responses, and underlying molecular mechanisms, may not faithfully recapitulate the *in vivo* situation. However, this cell line has been used in numerous studies to investigate receptivity and implantation (Wang et al., 2024; Ruane et al., 2024). Notwithstanding, our data reveals that the inhibitory effect of LEFTY2 on trypsin induced Ca^2+^ entry could not have been due to inhibition of ENaC, which is actually upregulated by LEFTY2 (Salker et al., 2016a). The trypsin induced [Ca^2+^]_i_ increase was strongly and significantly blunted by nifedipine. In the presence of nifedipine, LEFTY2 did not further modify trypsin induced increase of [Ca^2+^]_i_. Thus, LEFTY2 downregulated trypsin induced [Ca^2+^]_i_ increase is largely due to interference with nifedipine sensitive Ca^2+^ entry. Interestingly, there have been two clinical trials with the use of nifedipine prior to embryo transfers with the conjecture that a relaxed myometrium and vasodilation may serve to improve implantation rates. However, both studies showed a decrease in pregnancy rates compared with the placebo group. This observed negative effect may be due to blocking of the required rise in calcium necessary for implantation (Nataj Majd et al., 2022; Ng et al., 2019). We cannot rule the contribution of other calcium channels in the process of implantation and infertility. A study by Bahar et al. showed that there was a change in the methylation status and transcriptomic levels of several T-type calcium channels and was correlated with recurrent implantation failure (Davoodi et al., 2024). Additionally, progesterone is known to modulate calcium channels. According to our data (Supplementary Figure 5a) the addition of cAMP and MPA with and without LEFTY2 did not change (total) calcium entry in endometrial epithelial cells. Additionally, we also provide evidence that the store operated calcium entry (SOCE), NCX and NCKX are not involved in the effects of trypsin activity, pointing to a conserved role of L-type calcium channel function (Supplementary Figures 5b–d). Further work is required to validate whether LEFTY2 can alter additional transporters and ion channels in the endometrium.

Lifestyle factors contribute to obesity and metabolic syndrome which are known factors impairing endometrial function and decreasing fertility rates (Dag and Dilbaz, 2015; Vrhovac et al., 2021). Endometrial cells cannot synthesise glucose *de novo* and must take up glucose *via* transporters (Vrhovac et al., 2021). In the first stage of pregnancy, the endometrium provides nutritional support for the embryo, in a process known as histotrophic nutrition (Burton et al., 2002). Anaerobic glycolysis depends on the availability of glucose and valuable source of glucose is glycogen deposits within endometrial cells. During early development the embryo is dependent on anaerobic glycolysis for its energy supply, thereby a compromise in adequate storage may also result in poor pregnancy outcomes (Chen and Dean, 2023). The sodium-glucose transporter 1 (SGLT1 or SLC5A1) is a Na^+^-coupled glucose transporter responsible for taking up glucose against the electrochemical gradient and, thus glycogen storage in cells (Gyimesi et al., 2020). LEFTY2 can increase transcript levels and protein abundance of SGLT1 and glycogen abundance and may interfere with implantation and early pregnancy events (Zeng et al., 2020). Glucose uptake in endometrial cells is not solely dependent on SGLT1, but may also involve glucose transporters from the glucose transporter molecules (GLUT) family (Vrhovac et al., 2021). Studies have shown that GLUT1 protein levels are significantly reduced in endometrial biopsies from women with infertility (von Wolff et al., 2003). Obesity is associated with increased glycogen storage and also reduced fertility (Dag and Dilbaz, 2015). In keeping with this animal studies also suggest that high glucose levels are detrimental to endometrial function and lower fertility rates (Salker et al., 2017; Zhang et al., 2020). How altered LEFTY2 influences uterine glycogen metabolism and implantation is almost entirely unexplored and future studies are warranted.

Endometrial LEFTY2 appears to play a dual role in the regulation of Ca^2+^ entry. Firstly, the downregulation of Orai1 prevents the conversion of the endometrium into a receptive phenotype by attenuating the expression of Ca^2+^ sensitive receptivity genes (Salker et al., 2018). Secondly, LEFTY2 downregulates the effect of trypsin induced Ca^2+^ entry and could prevent embryo-induced Ca^2+^ entry. It is tempting to speculate that both Orai1 and SOCE are required for fine-tuning endometrial receptivity prior to embryo implantation and that trypsin-induced Ca^2+^ entry takes a leading role during embryo implantation (Ruan et al., 2012) suggesting that LEFTY2 is a potent inhibitor of both, SOCE and trypsin-induced Ca^2+^ entry.

**FIGURE 5:**
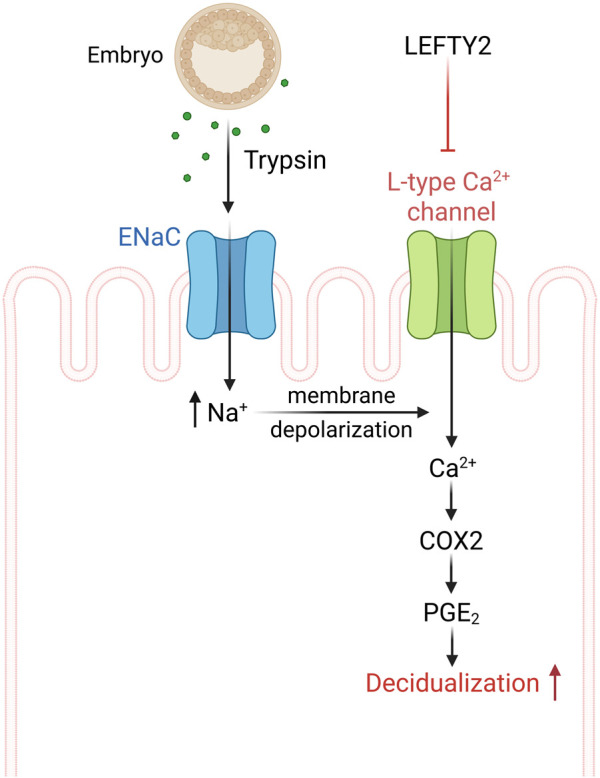
Schematic drawing of L-type calcium channel involvement in the process of implantation. ENaC activation by trypsin from the embryo causes epithelial cell membrane depolarization that activates L-type Ca^2+^ channel and Ca^2+^ influx. The endometrial epithelial Ca^2+^ influx can further activate cAMP-related pathways in stromal cells, leading to the process of implantation (Salker, 2025).

Taken together, our data demonstrates that embryo-derived trypsin is produced by human embryos and that increasing amounts are associated with a successful pregnancy. Our study further uncovers a negative influence of LEFTY2 on trypsin-induced nifedipine sensitive Ca^2+^ entry, an effect contributing to the adverse impact of LEFTY2 on embryo implantation (Figure 5).

## Data Availability

The raw data supporting the conclusions of this article will be made available upon request to MSS.
