# Supplementary material for: Embryo-derived trypsin-induced calcium entry is inhibited by endometrial infertility factor, LEFTY2
Source: Front Cell Dev Biol. 2025 May 29;13:1499339. doi: 10.3389/fcell.2025.1499339 (PMC12158927; doi:10.3389/fcell.2025.1499339)
Supplement: Supplementary file 1 [file Supplementaryfile1.docx]

**Supplementary Information**

**Embryo-derived trypsin-induced calcium entry is inhibited by endometrial infertility factor, LEFTY2.**

*Zhiqi Yang^1^, Jing Yan^2,3^, Steffen Kull^1^, Md Alauddin^1^, Sara Y. Brucker^1^, Melanie Henes^1^, Florian Lang^2^ and Madhuri S. Salker^1^*

^1^Women’s Health Research Institute, University Hospital Tübingen, Tübingen, Germany.

^2^Department of Physiology, University of Tübingen, Tübingen, Germany.

^3^Department of Physiology, Jining Medical University, Jining, PR China.


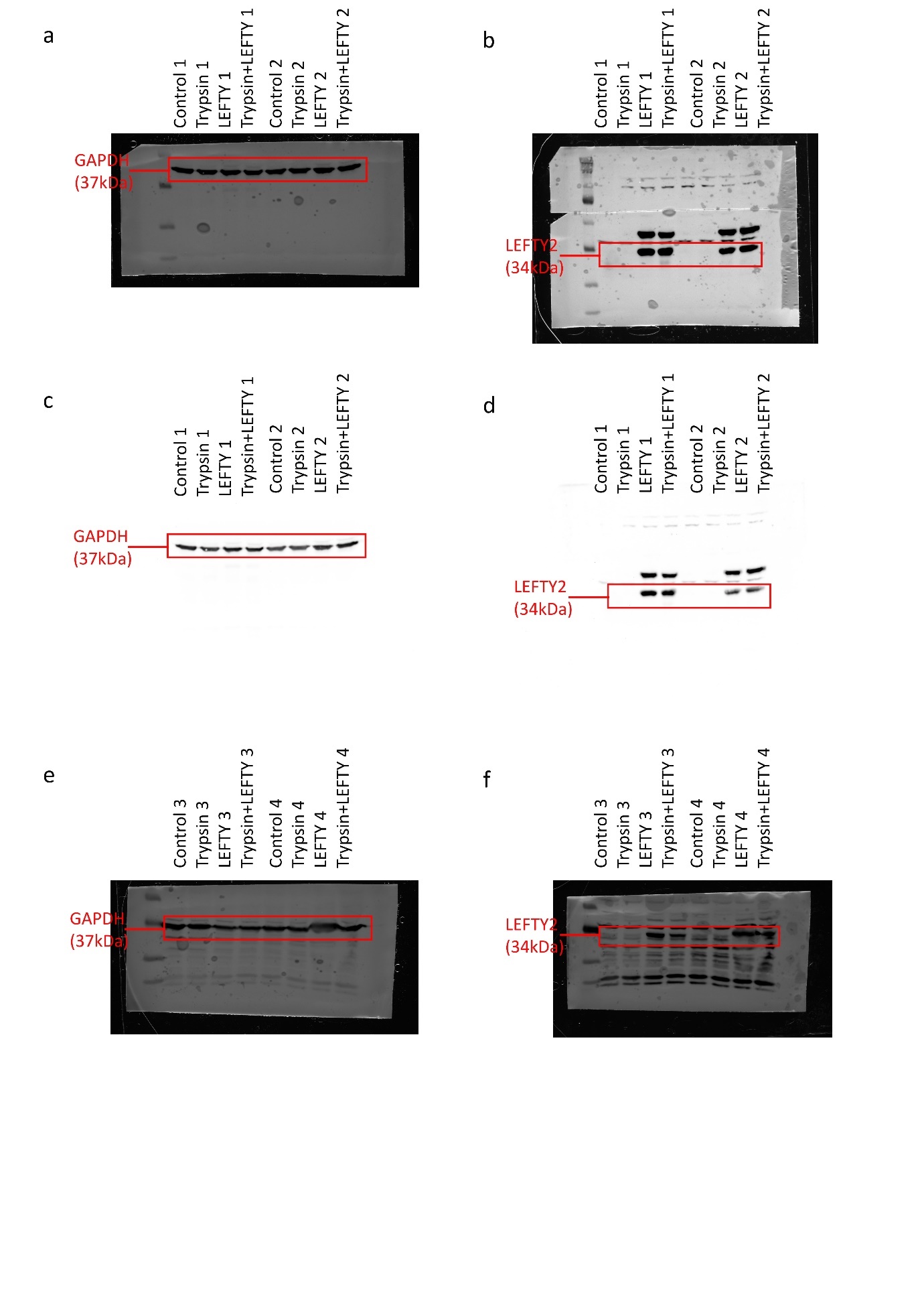


**Supplementary Figure 1: Original western blots used in Figure 3d.**

Original western blots with membrane overlay of (a, e) anti-GAPDH, 37kDa, and (b, f) anti-LEFTY2, 34kDa in Ishikawa cells treated with trypsin and LEFTY2 (Experiment 1-4). Original western blots (c, d) without membrane overlay were used in Figure 3d in the main file (Experiment 1).


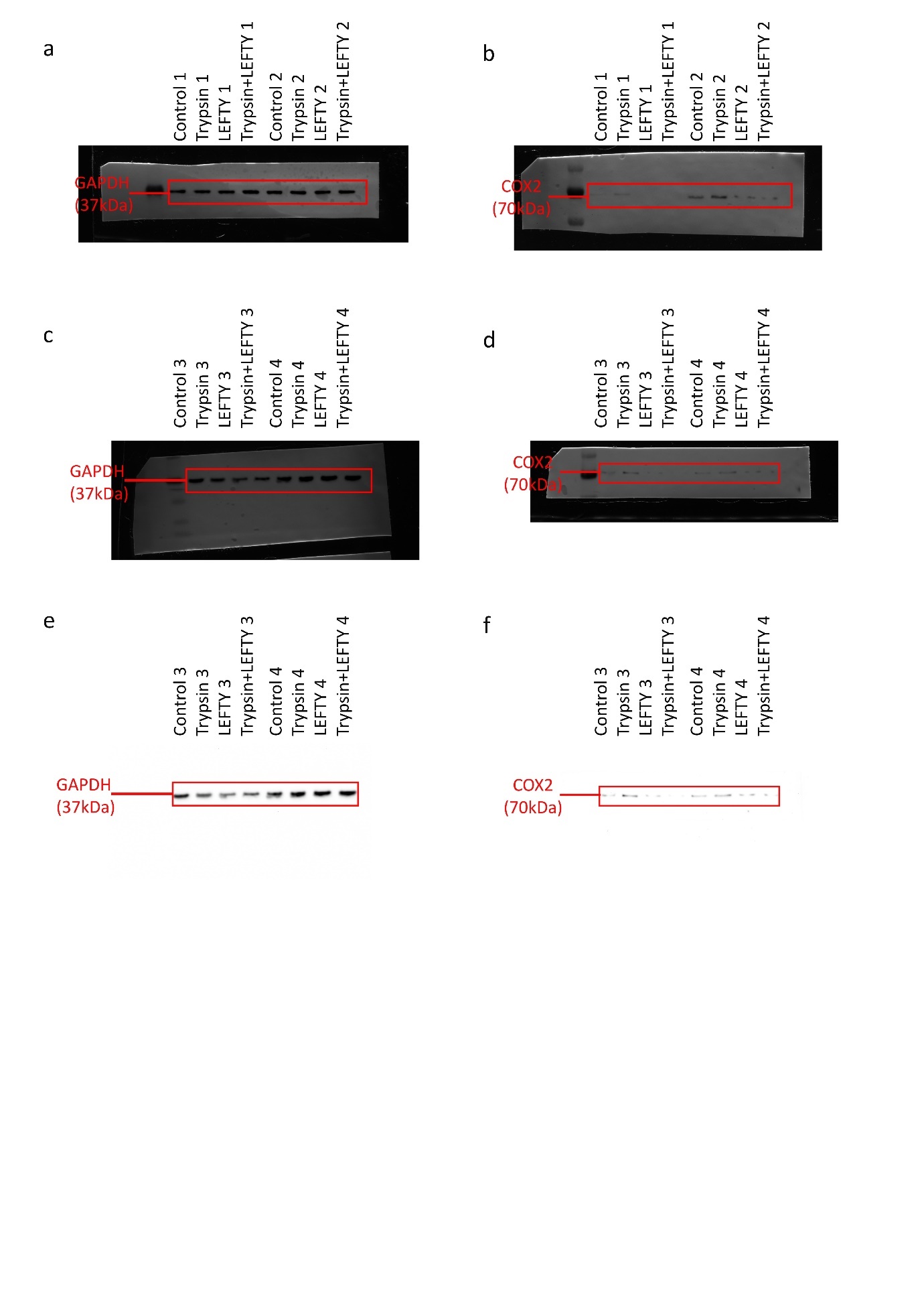


**Supplementary Figure 2: Original western blots used in Figure 3f.**

Original western blots of (a, c) anti-GAPDH, 37kDa, and (b, d) anti-COX2, 70kDa in Ishikawa cells treated with trypsin and LEFTY2 (Experiment 1-4). Original western blots (e, f) without membrane overlay were used in Figure 3f in the main file (Experiment 3).


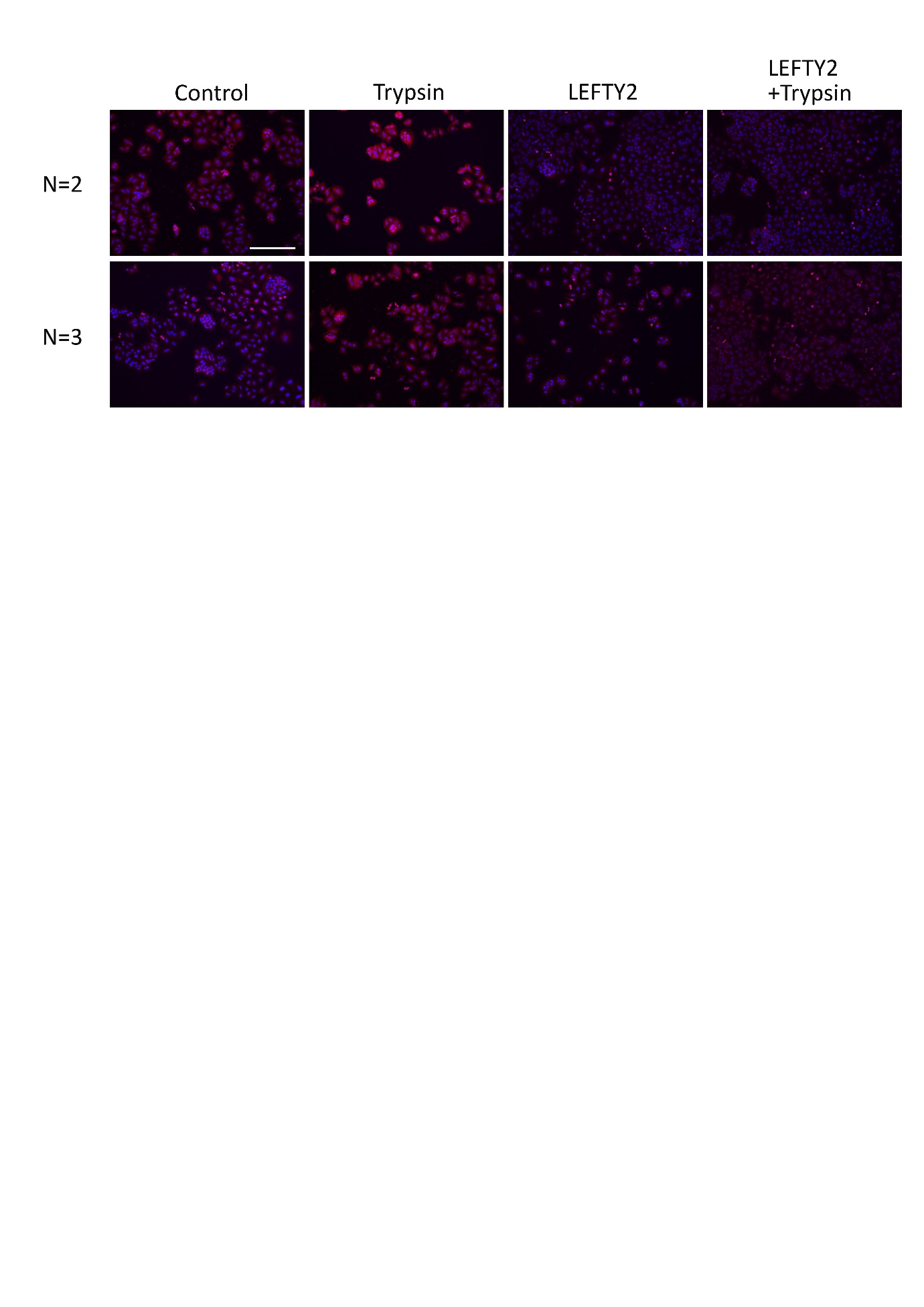


**Supplementary Figure 3: Additional images of CACNA1 immunofluorescence.**

Immunofluorescence microscopy of Ishikawa cells treated with or without LEFTY2 (25 ng/ml) for 6 h either in the presence or absence of Trypsin (20 µg/ml, 24h) showing CACNA1C subcellular localization in additional independent experiments. CACNA1C: Alexa Fluor 568 (red); nucleus: DAPI (blue). Scale bar=25µm.


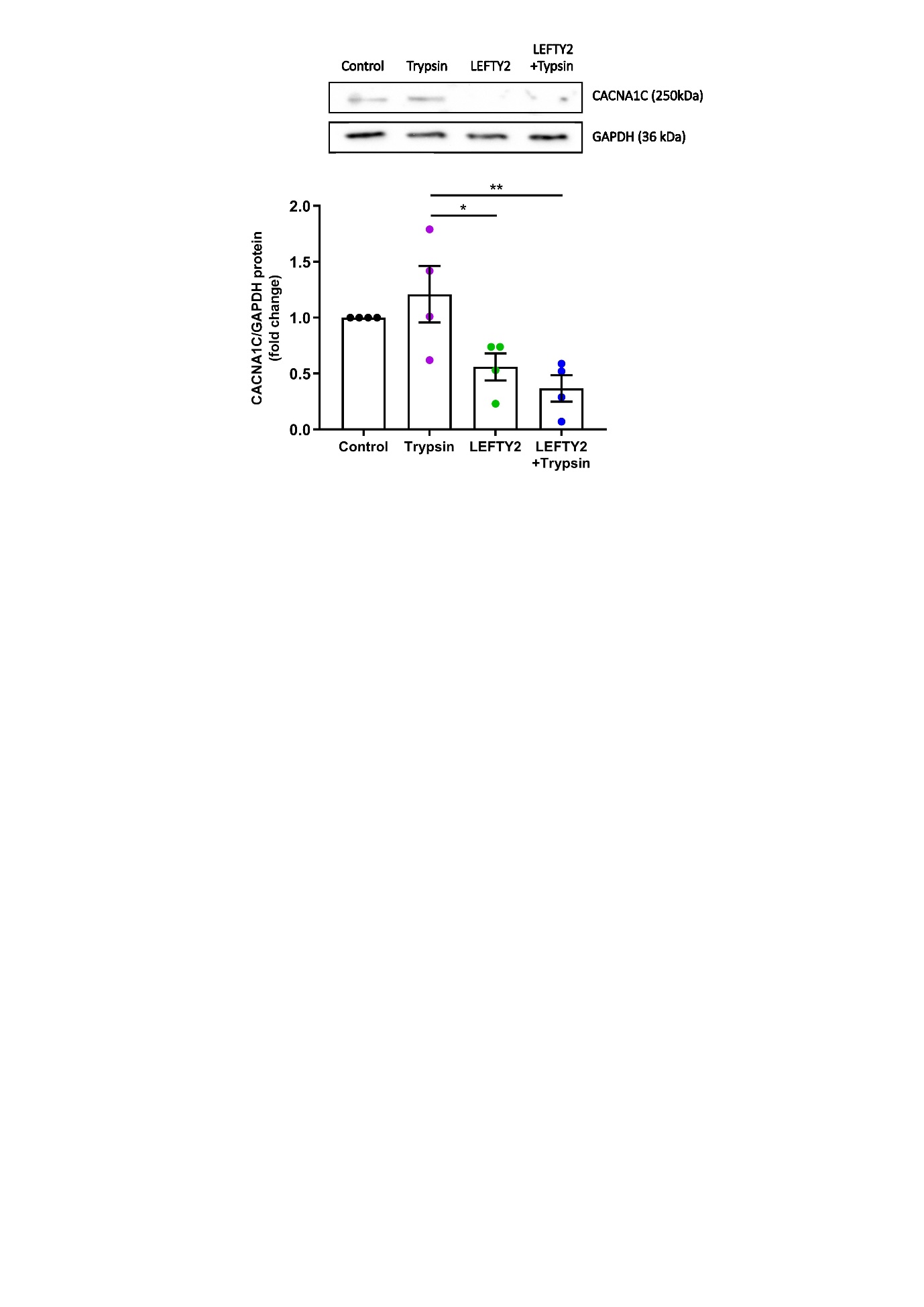


***Supplementary Figure 4: Western blot analysis of CACNA1C expression in Ishikawa cells.***

*Cell cultures were treated with or without LEFTY2 (25 ng/ml) for 6 h either in the presence or absence of Trypsin (20 µg/ml, 24h; n=4). GAPDH was used as a loading control. The data are presented as mean ± SEM. One-way ANOVA was used to calculate statistical significance. *P < 0.05, **P < 0.01, ***P < 0.001, ****P < 0.0001.*


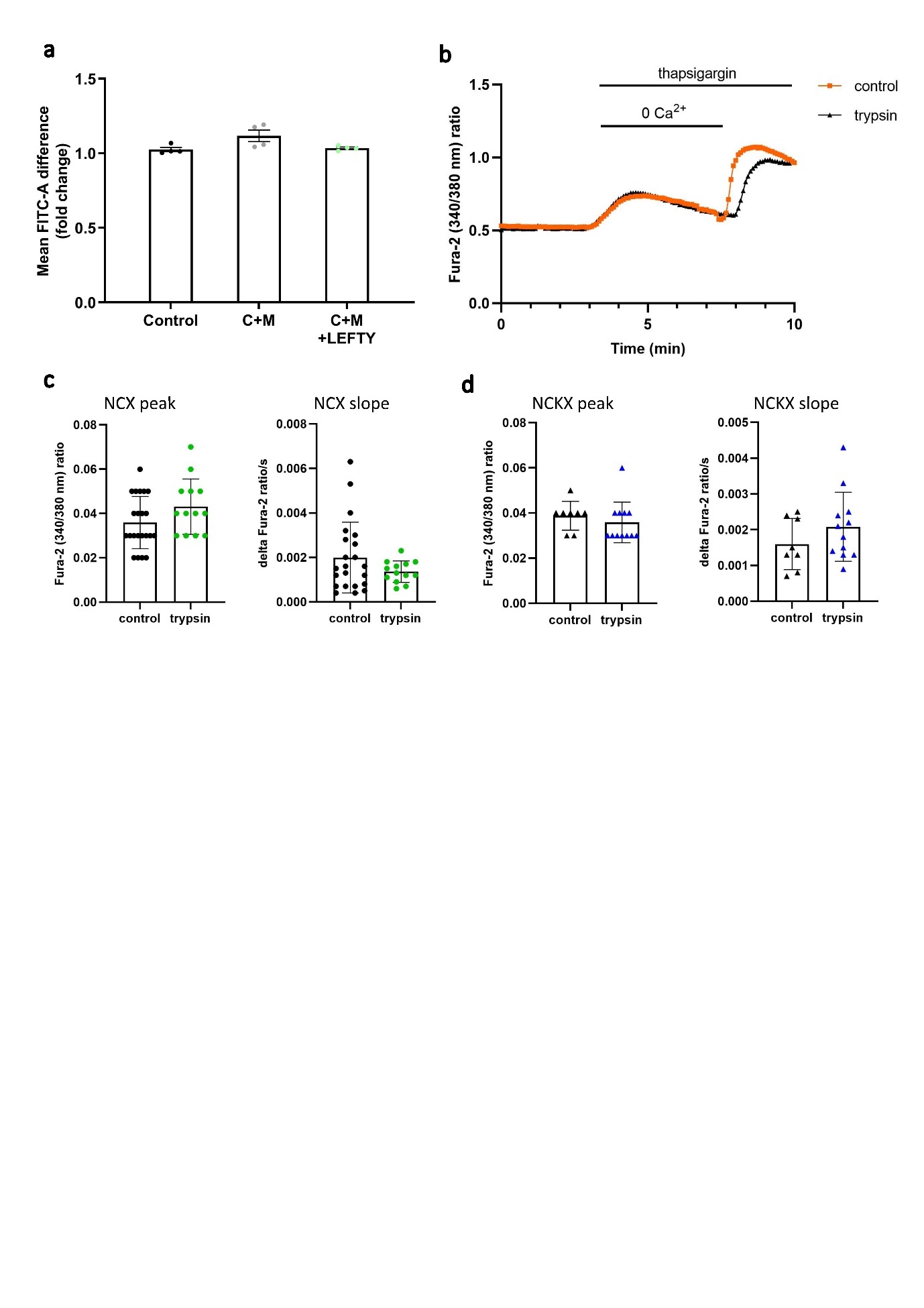


***Supplementary Figure 5: Effects of LEFTY2 and Trypsin on Calcium Entry in Ishikawa Cells.***

***(a)*** *Ishikawa cells were treated with cAMP (0.5 µM) and MPA (1µM) for 6 days, followed by treatment with or without LEFTY2 (25 ng/ml) for 6 h either in the presence or absence of Trypsin (20 µg/ml, 24h). Fluorescence-activated cell sorting analysis (FACS) (n=4) of trypsin-induced calcium entry in Ishikawa cells. The FITC-A difference was calculated as the ratio of the FITC-A intensity of the cells after trypsin addition to that before trypsin addition.* ***(b)*** *Original tracing of Fura-2 fluorescence-ratio in fluorescence spectrometry during and after Ca^2+^ depletion (1 μM thapsigargin) in trypsin treated (black triangles) and vehicle treated (PBS; orange squares) Ishikawa cells.* ***(c)*** *Arithmetic means ± SEM (n = 3, each experiment 3-10 cells) of the peaks (left panels) and slopes (right panels) of NCX-mediated Ca^2+^ entry.* ***(d)*** *Arithmetic means ± SEM (n = 3, each experiment 3-10 cells) of the peaks (left panels) and slopes (right panels) of NCKX-mediated Ca^2+^ entry. The data are presented as mean ± SEM. One-way ANOVA and unpaired student’s t-test were used to calculate statistical significance. *P < 0.05, **P < 0.01, ***P < 0.001, ****P < 0.0001.*
